# Mapping the Temporal Landscape of Breast Cancer Using Epigenetic Entropy

**DOI:** 10.21203/rs.3.rs-5119308/v1

**Published:** 2024-10-28

**Authors:** darryl shibata, Daniel Monyak, Shannon Holloway, Graham Gumbert, Lars Grimm, Shelley Hwang, Jeffrey Marks, Marc Ryser

**Affiliations:** USC School of Medicine; Duke University; Duke University; Duke University; Duke University; Duke University; Duke University; Duke University

## Abstract

Although generally unknown, the age of a newly diagnosed tumor encodes valuable etiologic and prognostic information. Here, we estimate the age of breast cancers, defined as the time from the start of growth to detection, using a measure of epigenetic entropy derived from genome-wide methylation arrays. Based on an ensemble of neutrally fluctuating CpG (fCpG) sites, this stochastic epigenetic clock differs from conventional clocks that measure age-related increases in methylation. We show that younger tumors exhibit hallmarks of aggressiveness, such as increased proliferation and genomic instability, whereas older tumors are characterized by elevated immune infiltration, indicative of enhanced immune surveillance. These findings suggest that the clock captures a tumor’s effective growth rate resulting from the evolutionary-ecological competition between intrinsic growth potential and external systemic pressures. Because of the clock’s ability to delineate old and stable from young and aggressive tumors, it has potential applications in risk stratification of early-stage breast cancers and guiding early detection efforts.

## INTRODUCTION

When a woman is diagnosed with breast cancer, it is generally not possible to ascertain how long the tumor has been growing. Yet knowledge about a tumor’s age at diagnosis could provide important prognostic clues: older indolent tumors that are less likely to progress may require less invasive treatment, whereas younger fast-growing tumors require more urgent and aggressive treatment. While there is a rich literature on the estimation of the mean sojourn time of breast cancer ^1–5^–the average time tumors spend in a detectable but asymptomatic, pre-clinical state–there is a paucity of tools to assess the age of individual tumors at the time of detection.

Epigenetic clocks provide a promising approach to estimate individual tumor age. Originally developed to quantify the biologic aging process in humans, epigenetic clocks leverage specific patterns of DNA methylation that are strongly correlated with biologic tissue age. ^6,7^ Broadly, these clocks focus on the methylation status of CpG sites across the genome that are unmethylated at birth and become methylated with increasing tissue age due to the accumulation of stochastic replication errors. Due to a low error rate, such clocks are well suited to resolve processes that evolve over time scales on the order of decades, such as normal tissue aging and pre-neoplastic transformations. ^8–12^ They are, however, less useful for estimating the age of invasive cancers, which take mere months to years to become symptomatic or reach a screen detectable size.

**Figure 1.**
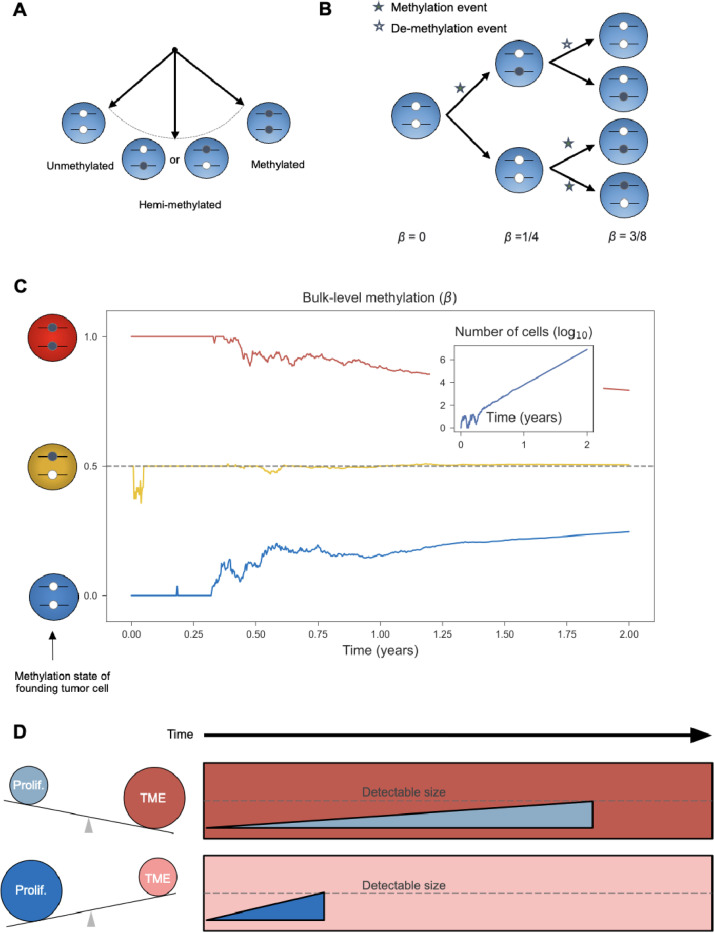
Fluctuating CpG sites (fCpG) and modulators of tumor age. **(A)** Balanced fCpGs stochastically oscillate between the unmethylated, hemi-methylated, and methylated states. **(B)** During tumor cell division, methylation and demethylation events occur stochastically. As illustrated for the first three generations of an exponential tumor expansion, the average methylation status (β-value) evolves over time. **(C)** In silico modeling of the fCpG dynamics, based on a simple birth-death model of tumorigenesis. The average methylation value (β) is simulated for three independent fCpGs, whose initial states in the first tumor cell are fully methylated (red), hemi-methylated (yellow), and unmethylated (blue), respectively. Insert: the number of tumor cells over time. Simulation parameters: cell proliferation rate α=0.17 divisions/day; cell death rate λ=0.15 deaths/day; (de-)methylation rate μ=0.002 flips/division. See Methods for details. **(D)** The age of a tumor at detection is modulated by relative strengths of intrinsic proliferation and growth-suppression induced by the immune tumor microenvironment (TME); tumors will reach a detectable size faster when proliferation is greater and/or the TME is weaker.

In contrast to “one-way” clocks that measure age-related increases of methylation, “two-way” epigenetic clocks leverage CpG sites that fluctuate between the unmethylated and methylated states on a relatively fast time scale (Fig. 1A). Originally introduced in the context of homeostatic intestinal stem cell dynamics, ^13^ such stochastic epigenetic clocks have also been applied to hematologic malignancies. ^14^ Here we follow similar design principles to develop a breast cancer-specific two-way epigenetic clock to measure individual tumor age at diagnosis based on average methylation levels (β-values) of select CpG sites included in standard methylation arrays.

The proposed stochastic clock measures the entropy of an ensemble of fluctuating CpG (fCpG) sites. In the tumor’s most recent common ancestor cell, each f pG was either unmethylated (β=0), hemi-methylated (β=0.5), or methylated (β=1). As the tumor expands, replication errors produce a mixture of cells with different methylation states (Fig. 1B), thus progressively increasing the tumor’s epigenetic entropy. ^15^ In the special case of unbiased fCpG sites-whose methylation and demethylation rates are in balance-the bulk-level methylation converges to β=0.5 with increasing tumor age, regardless of the first tumor cell’s state (Fig. 1C). Thus, by measuring the distribution of unbiased fCpG sites, we can derive an estimate of the age of a given tumor cell population relative to the start of the most recent clonal expansion.

The combination of tumor-specific age estimates and gene expression profiles further provides a unique opportunity to characterize the evolutionary and ecological pressures that shape the temporal landscape of breast cancer. Notably, aggressive tumors that evolve in a weakly suppressive immune microenvironment are expected to reach a detectable size faster than indolent, slow-growing tumors in a strongly suppressive immune microenvironment (Fig. 1D).

The manuscript is structured as follows. Combining DNA methylation and gene expression data from several hundred breast cancer and normal breast tissue samples, we first identify a set of unbiased fCpG sites and introduce the epigenetic clock index as a proxy measure of tumor mitotic age. We then evaluate the face validity of the index by examining its relationship with established prognostic markers, and we combine methylation and gene expression data to identify tumor- and microenvironment-specific factors that modulate tumor age. Finally, we validate key properties of the clock index in independent cohorts of patients with paired primary-metastasis samples, and we derive quantitative estimates of individual breast cancers’ mitotic and calendar ages.

## RESULTS

### Selection of unbiased fCpG sites

To identify a set of unbiased fCpG sites in breast cancer, we used 450K methylation array data from 634 invasive breast cancers in The Cancer Genome Atlas ^16^ (TCGA) and 79 normal breast tissue samples. ^17^ Using a three-step selection process, we identified an ensemble of fCpG sites with balanced (de-)methylation rates as follows.

**Figure 2.**
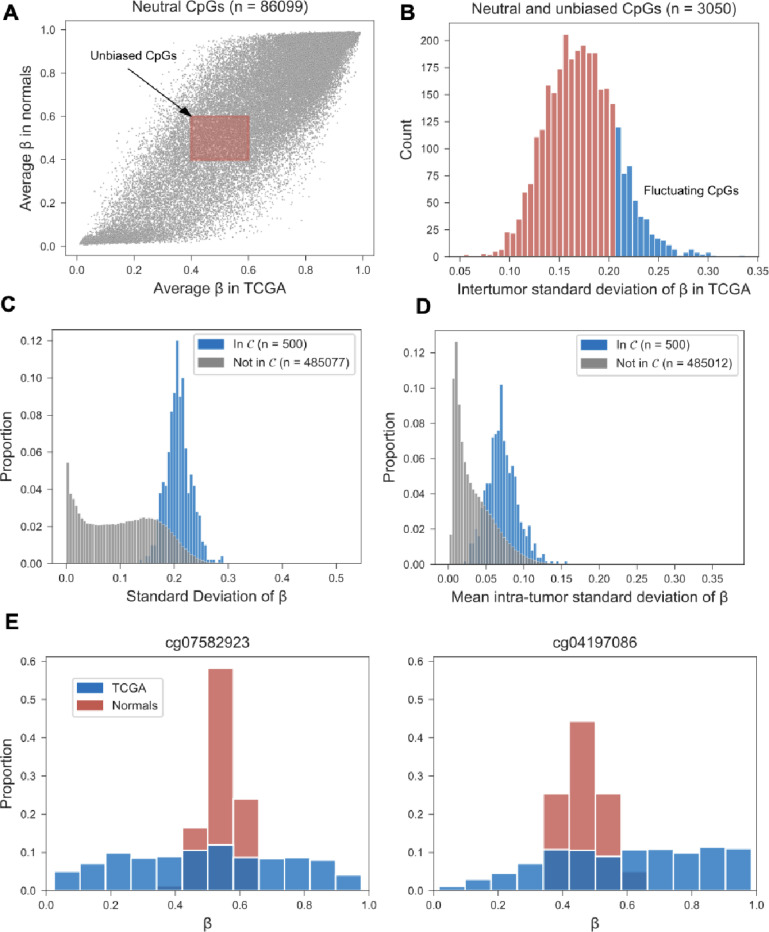
Selection and validation of unbiased fluctuating CpG (fCpG) sites. **(A)** Starting with CpG sites that are nonregulatory and non-genic (gray dots), sites with an average methylation value β between 0.4 and 0.6 in both breast cancers (N=634, TCGA cohort) and normal samples (N=79, Normal cohort) were considered unbiased (red shaded rectangle). **(B)** Sites were ranked by their inter-tumor standard deviation, and the top 500 were included the clock set C. **(C)** In a separate cohort of breast cancers (N=146, Lund cohort), the inter-tumor standard deviation of β was higher for fCp G sites in the clock set C (median: 0.206) compared to CpGs not included in C (median: 0.102; P=1×10^−238^, Wilcoxon rank-sum test). **(D)** In a small cohort of patients (N=5) with multiple primary samples (3–5 per patient), the intra-tumor standard deviation of β was higher for CpG sites in the clock set C (median: 0.070), compared to CpGs not included in C (median: 0.026; P=6 0×10^−179^, Wilcoxon rank-sum test). **(E)** Distribution of the β-values of two fCpG sites in the clock set C, across breast cancers (N=634, TCGA cohort) and normal breast samples (N=76, Normal cohort).

In the first step, we eliminated regulatory and genic loci due to their increased likelihood of being under selection during tumor growth. This choice contrasts with the majority of cancer epigenomic studies focused on functional CpG sites located in or around promoter regions. ^17–19^ Next, we identified CpG sites with an average β-value close to 0.5 in both normal breast tissue and breast cancers, thus excluding sites with an inherent bias toward methylation or de-methylation, and sites that are subject to systematic selection during homeostasis and/or tumorigenesis (Fig. 2A). In the third step, we ordered the set of unbiased CpG sites by between-tumor variability and included only the 500 most fluctuating sites in the final clock set of unbiased fCpGs (Fig. 2B). Importantly, this final step excludes non-informative sites that either do not fluctuate at all (i.e., imprinted hemi-methylated state) or fluctuate too fast (i.e., steady-state methylation of β≈0.5 reached on time scales much shorter than the average mitotic tumor age at diagnosis).

Next, we sought to validate the unbiased and fluctuating nature of the clock set in two independent cohorts. In a cohort of 146 breast cancer patients (Lund cohort), ^20^ we found significantly higher inter-tumor variability in β-values among the CpG sites in the clock set, as compared to the CpG sites not included in the clock set (Fig. 2C). Similarly, in a small cohort of 5 patients with multiple samples from their primary tumors, ^21^ we found elevated intra-tumor variability in clock set vs non-clock set sites (Fig. 2D). Together, these patterns corroborate the unbiased and fluctuating nature of the clock set of CpG sites.

Interestingly, the fCpG sites in the clock set were more tightly concentrated around β=0.5 in normal breast tissue, as compared to breast cancers (Fig. 2E), with a median standard deviation of β-values in normal samples of 0.09, compared to 0.21 in tumors (P = 2×10^−45^, Wilcoxon rank sum test). Consistent with the underlying dynamic model of the clock (Fig. 1A), this suggests that over decades of breast development and maintenance, the fCpGs had converged to the stationary methylation state of β=0.5 (Fig. 1C).

### Epigenetic clock index

**Figure 3.**
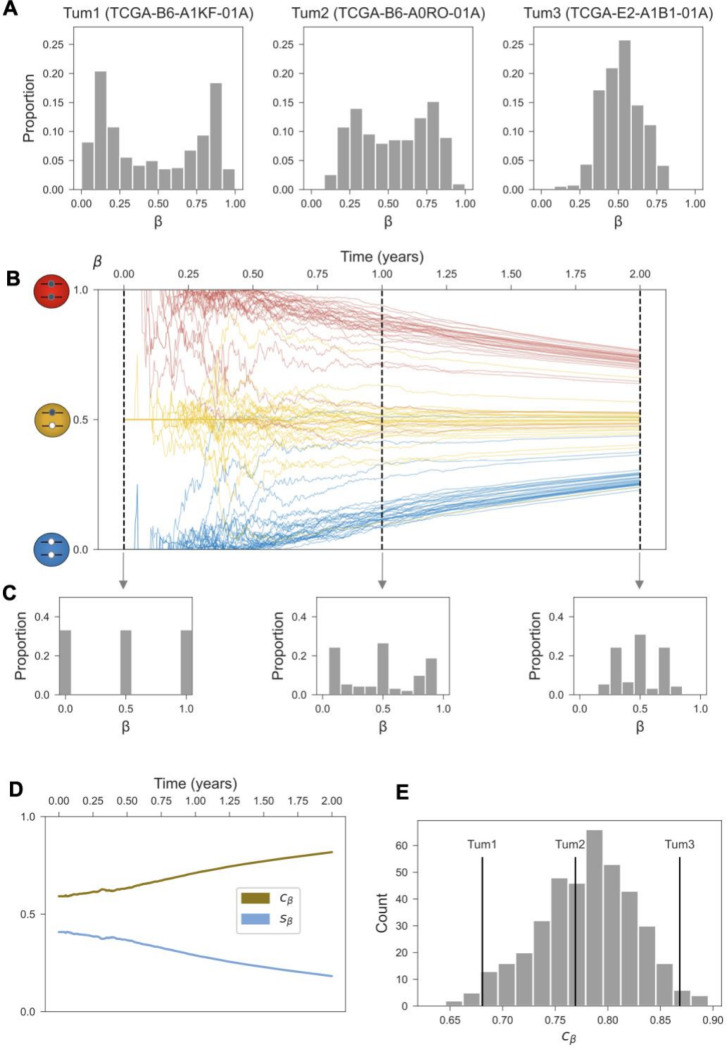
The distribution β-values across the clock set encodes tumor age. **(A)** The empirical β-value distributions for the clock set fCpGs (N=500) are shown for three select tumors in the TCGA cohort. **(B)** Simulated trajectories for an ensemble of fCpG sites (N=90), starting in the unmethylated, hemi-methylated, and methylated initial configurations, respectively (n = 30 each). The thick lines represent the average β-value trajectories for each subset. Simulation parameters as detailed in Figure 1. **(C)** Cross-sectional β-value distributions for the simulated clock set in panel B, shown after 0,1, and 2 years of growth. **(D)** Standard deviation ($s_{\beta }$) of β-values and epigenetic clock index ($c_{\beta }=1-s_{\beta }$) over 2 years of growth for the simulated clock set in panel B. **(E)** The distribution of epigenetic clock index values $\left(c_{\beta }\right)$ across invasive ductal carcinomas in TCGA (N=400); the three tumors from (A) are labeled.

At the level of individual tumors, the 500 fCpG sites in the clock set exhibited primarily unimodal or bimodal distributions of β-values (Fig. 3A). We explored how these tumor-specific distributions of β-values could be used to estimate tumor mitotic age. In the founding tumor cell, each fCpG starts in either the unmethylated (β=0), hemi-methylated (β=0.5), or methylated (β=1) state (Fig. 1A). Although the trajectories of individual sites are subject to stochastic fluctuations (Fig. 1C), an ensemble of sites starting in the same initial configuration collectively drift toward the steady state of β=0.5 (Fig. 3B).

By considering the histograms of β-value distributions at different mitotic ages, we can track the evolution of the three “peaks” corresponding to the subsets of initially unmethylated, hemi-methylated, and methylated clock sites (Fig. 3C). As the tumor’s mitotic age increases, the left peak of the histogram (consisting of originally unmethylated clock sites) starts moving to the right, whereas the right peak (originally methylated sites) moves to the left; the middle peak (originally hemi-methylated sites) remains stationary. By measuring the extent to which the three peaks have converged to the stationary value of β=0.5, we can thus estimate the mitotic age of individual tumors.

Concretely, we used the standard deviation of the β-values, denoted by $s_{\beta }$, to quantify the relationship between mitotic tumor age and the evolving clock set profile (Fig. 3C). Because $s_{\beta }$ is highest at time 0, when the β-value distribution exhibits three sharp peaks, and then monotonically decreases over time (Fig. 3D), we introduced the epigenetic clock index $c_{\beta }=1-s_{\beta }$ as a proxy measure of mitotic tumor age (Fig. 3E).

In the next two sections, we characterize the relationship between a tumor’s mitotic age, as quantified by the epigenetic clock index $c_{\beta }$, and its evolutionary-ecological context as determined by its intrinsic growth potential and external pressures from the microenvironment. Because there is a pronounced difference in $c_{\beta }$ between ductal carcinomas (median, 0.79) and lobular carcinomas (median, 0.82; P = 2×10^−16^, Wilcoxon rank-sum test), we restricted subsequent analyses to the more common ductal carcinomas to avoid unnecessary confounding.

### Younger tumors have more aggressive phenotypes

As a breast tumor grows, its likelihood of detection on the basis of imaging or symptoms increases. Because fast growing tumors are expected to reach a detectable size sooner than slow growing ones, we hypothesized that younger tumor mitotic age would correlate with established markers of tumor aggressiveness. To test this hypothesis, we correlated the epigenetic clock index with several established features of tumor aggressiveness, including molecular subtype, ^22,23^ genomic instability, ^24,25^ grade, ^26^ and size. ^27^

**Figure 4.**
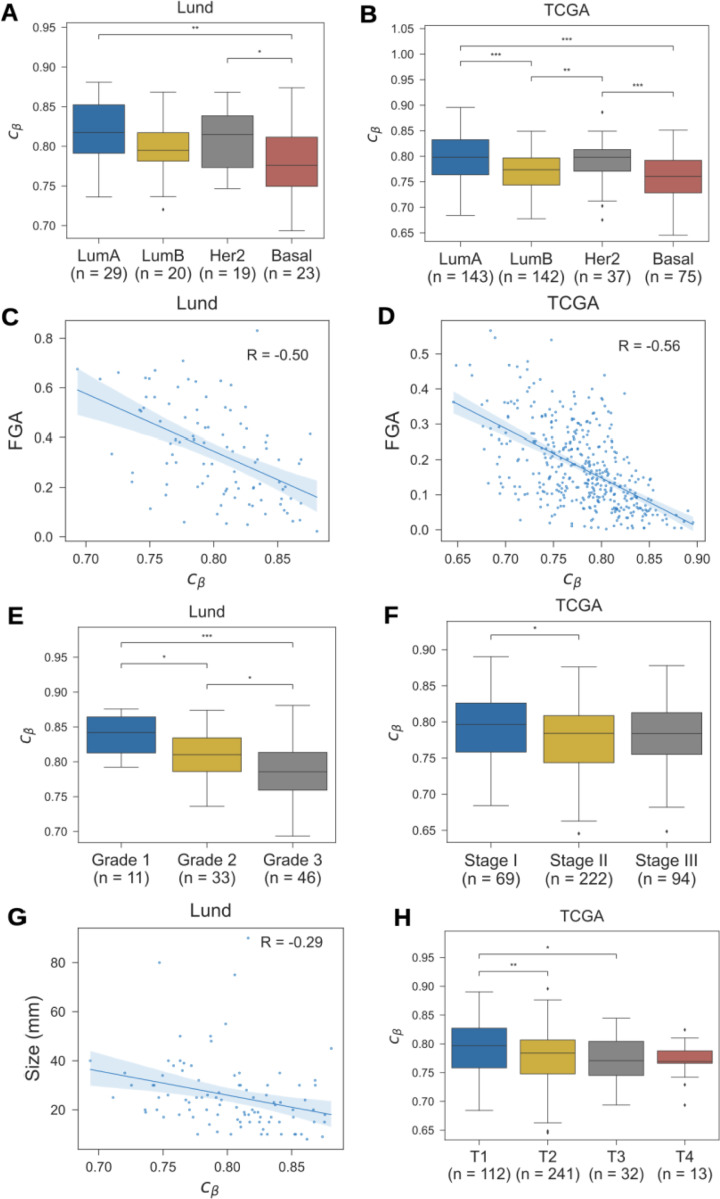
Epigenetic clock index vs. clinicopathological variables. The distribution of epigenetic clock index values ($c_{\beta }$) among invasive ductal carcinomas in the Lund and TCGA cohorts, by **(A)** tumor grade; **(B)** tumor stage; **(C)-(D)** molecular subtype as predicted by the PAM50 algorithm; **(E)** tumor size; **(F)** T-stage; and **(G)-(H)** fraction of genome altered (FGA) by copy number alterations. Pairwise comparisons of medians in panels A,B,E,F, and H were performed using a two-sided Wilcoxon rank-sum test (*P < 0.05,**P < 0.01,***P < 0.001). In panels C, D and G, regression lines and bootstrapped 5% confidence intervals are shown; Pearson correlations (R) are indicated.

There was a clear relationship between mitotic age and molecular subtype: luminal A tumors, which have a more favorable prognosis, were older than luminal B and basal tumors (Figs. 4A-B; Suppl. Table S1). Similarly, there was a strong correlation between genomic instability and younger tumor age (Figs. 4C-D), and younger tumors were of higher histopathologic grade (Fig. 4E). In contrast, there was only a weak relationship between mitotic age and summary stage (Fig. 4F)

Another prognostic factor in breast cancer is tumor size, with larger lesions having worse outcomes. We found that smaller tumors were of older mitotic age compared to larger tumors (Figs. 4G-H), presumably because slow growing tumors spend more time at the smaller end of the detectable size range, and are, therefore, more likely to be detected at a smaller size.

Finally, the relationship between tumor mitotic age and patient age at diagnosis was inconclusive, with a weak negative correlation in TCGA (*R*=−0.18) and no correlation in the Lund cohort (*R*=−0.06; Suppl. Table S1). This is consistent with the notion that the fCpG clock measures the age of the tumor-starting with the most recent common ancestor cell-and not the age of the patient.

### Identifying modulators of mitotic tumor age

**Figure 5.**
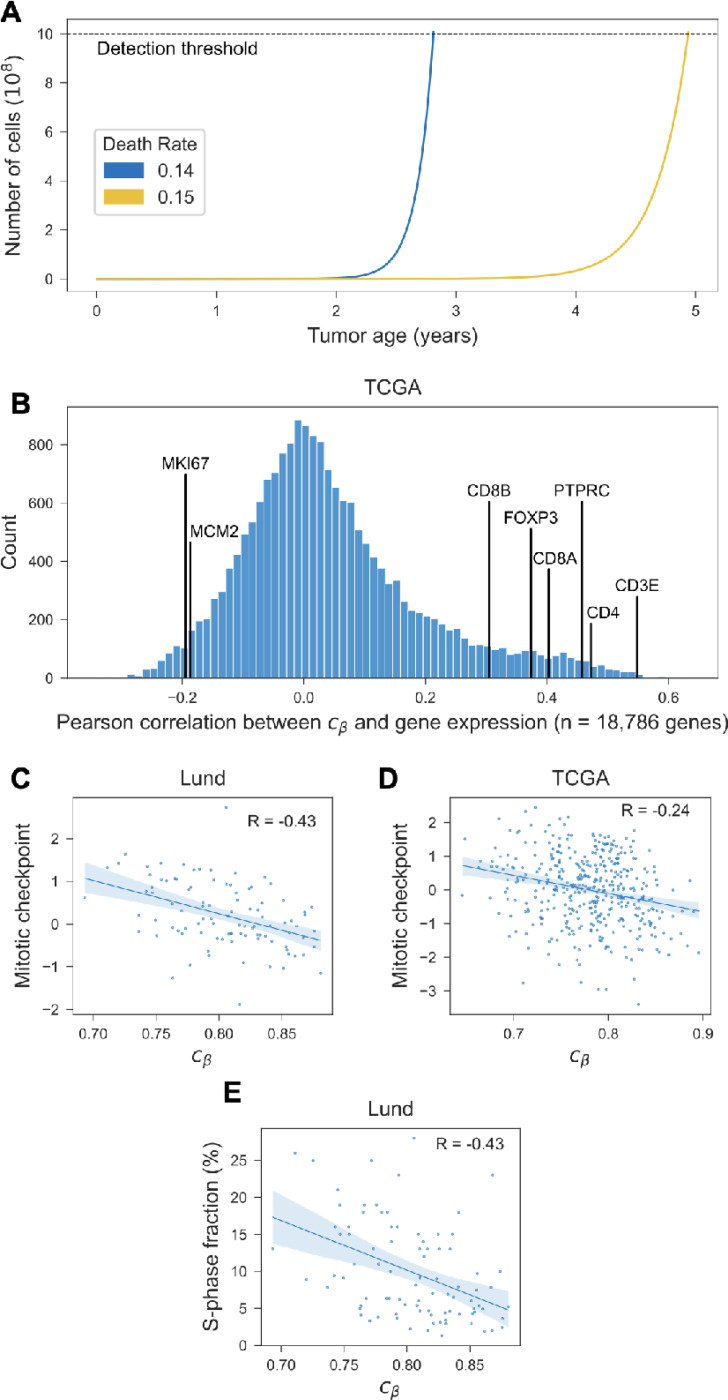
Mitotic tumor age vs. measures of proliferation. **(A)** Simulation of tumor size as a function of tumor age. Both tumors have the same proliferation rate (α=0.16 divisions/day), but different death rates: λ=0.141/ day (blue) vs. λ=0.15 1/day (yellow). **(B)** Pearson’s correlation between epigenetic clock index $c_{\beta }$ and expression of protein-coding genes (TCGA cohort); correlation with select genes as indicated. **(C)-(D)** Correlation of $c_{\beta }$ with average expression of genes involved in M-phase and mitotic checkpoint regulation. **(E)** Correlation of $c_{\beta }$ with the fraction of cells in S-phase, as measured by flow cytometry (Lund cohort). Regression lines shown with bootstrapped 95% confidence intervals and Pearson correlation (R).

The time it takes for a tumor to grow from a single cell to a detectable mass depends on its effective growth rate, that is the difference between cell proliferation and cell death (Fig. 5A). Cell proliferation primarily reflects the tumor’s intrinsic growth potential and aggressiveness, whereas cell death is often the result of extrinsic selective pressures applied by the tumor microenvironment, such as immune surveillance and resource constraints due to limited vascularization. ^28,29^

To explore putative modulators of effective tumor growth and mitotic age at diagnosis, we performed genome-wide correlation analyses of the epigenetic clock index $c_{\beta }$ against gene expression. As predicted, mitotically younger tumors exhibited increased expression of proliferation-related genes such as Ki67 and MCM2 (Fig. 5B, Suppl. Table S1). The signal was further augmented when considering the average expression across a set of genes involved in M-phase and mitotic checkpoint regulation (Fig. 5C-D) and the fraction of cells in S-phase (Fig. 5E).

Next, we examined the microenvironment’s ability to decrease the effective growth rate of a tumor through increased cell death. As hypothesized, the expression of immune cell markers such as CD3, CD4, CD8 and FOX3 was elevated in mitotically older tumors (Fig. 5B; Suppl. Table S1). This suggests that tumors which are subject to immune surveillance-e.g., through neo-antigen directed immune control by CD8 + T-cells-have a lower effective growth rate and, thus, reach a detectable size at an older mitotic age, as compared to tumors that successfully evade immune control and thus reach a detectable size at a younger mitotic age.

**Figure 6.**
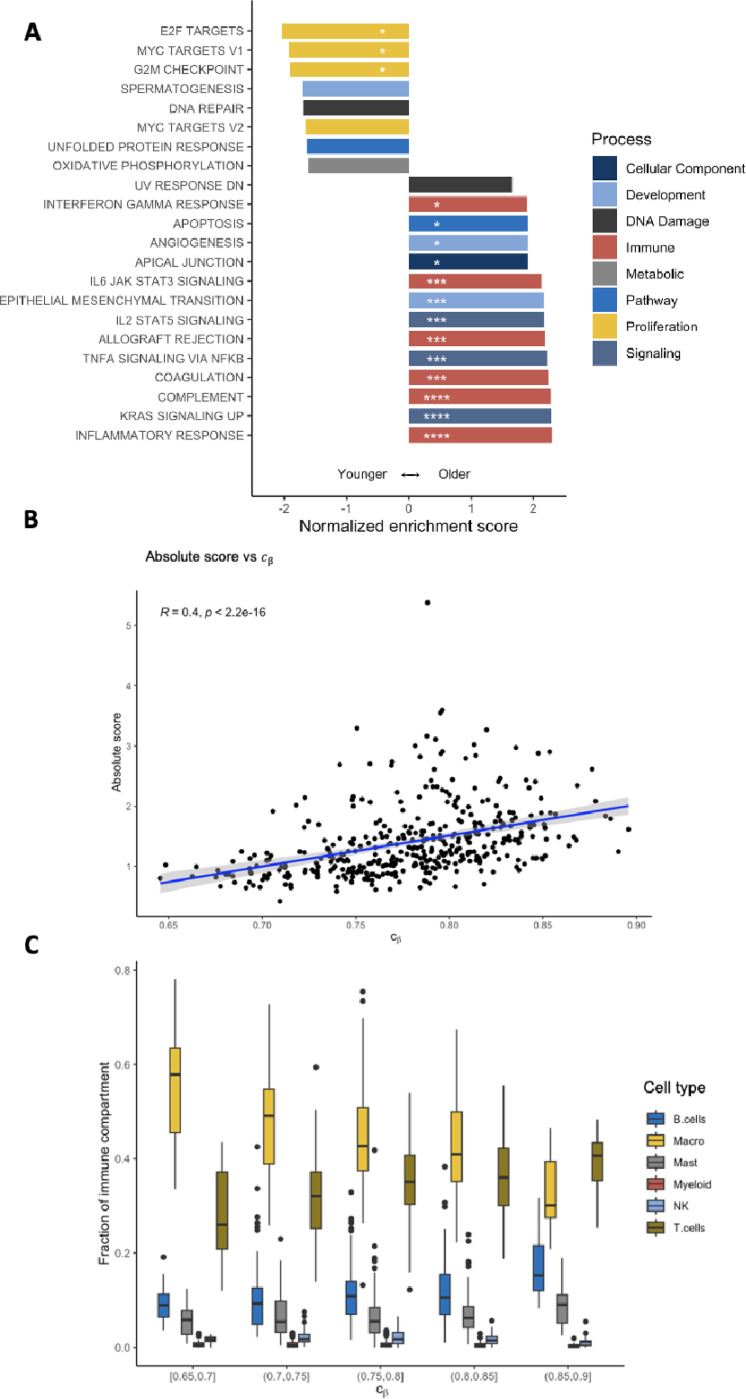
Pathway enrichment and immune decomposition analyses. **(A)** A gene set enrichment analysis (GSEA) was performed for the epigenetic clock index $c_{\beta }$. Pathways with a positive (negative) enrichment score are enriched in mitotically older (younger) tumors. Only pathways with a false discovery rate (FDR) below 0.1 are shown; *FDR<.05; **FDR<.01; ***FDR<.001; ***FDR<.0001. **(B)** Epigenetic clock index vs. the extent of immune infiltration (absolute immune score) as estimated by CIBERSORTx. **(C)** The immune compartment of each tumor was decomposed using CIBERSORTx; the compartment fractions are shown for tumors of similar mitotic age (epigenetic clock index $c_{\beta }$).

To perform a systematic analysis of mitotic tumor age modulation, we performed a genome-wide gene set enrichment analysis (GSEA) (Fig. 6A). Consistent with the univariate gene expression analyses, mitotically younger tumors were enriched for pathways related to proliferation and cell cycle control. Conversely, mitotically older tumors were enriched for immune pathways and immune-related signaling pathways, again supporting the notion of effective immune control in older, slower growing lesions.

For a more in-depth analysis of the immune infiltrate, we used the CIBERSORTx algorithm ^30^ to estimate the extent and composition of the immune compartment. As expected, the extent of the immune compartment increased with mitotic tumor age (Fig. 6B). When decomposing each tumor’s immune compartment into the major cell types, we found an increase in the fraction of T-cells in mitotically older tumors (Fig. 6C, Suppl. Table S2), again suggestive of T-cell mediated immune surveillance.

### Analysis of paired tumor samples validates epigenetic clock

**Figure 7.**
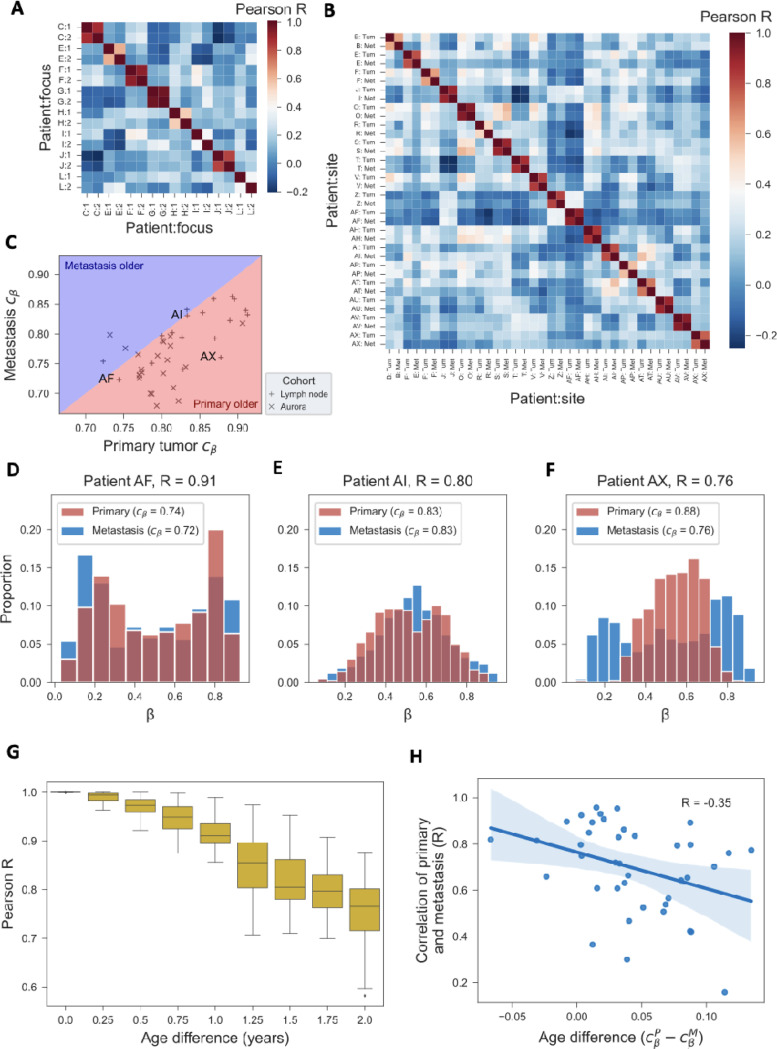
Paired tumor samples. **(A)** In a cohort of 8 patients with multifocal breast cancer, the β-values of the 500 fCpG sites of the epigenetic clock are correlated within (two foci per patient) and between patients. **(B)** In a cohort of 18 patients with paired primary tumor and lymph node metastasis samples, the β-values of the 500 fCpG sites are correlated within and between patients. **(C)** For the 18 patients from panel B and 22 patients from the AURORA US Metastasis Project, the epigenetic clock index of the primary tumor is plotted against the index of the lymph node metastasis. Three patients (AF, Al, and AX) from the lymph node cohort are labeled for the purposes of the following 3 panels. **(D-F)** For the 3 labeled patients from panel C, the β-value distributions of fCpGs are shown both for the primary tumor and the metastasis. **(G)** Monte Carlo simulation of an ensemble of 90 fCpG sites, 30 each starting in the unmethylated, hemi-methylated, and methylated initial configurations; every 3 months, a cell was randomly picked to representing the metastasis seeding cell, and the Pearson correlation between the β-value distribution of that cell and that of the entire tumor was calculated. Distributions at each time point represent the results from 30 independent simulations. Simulation parameters are detailed in Figure 1. **(H)** For the 18 patients from panel B and 22 patients from the AURORA US Metastasis Project, the epigenetic relatedness of primary and lymph node metastasis (Pearson’s R for the 500 fCpG sites in the clock set) is compared to the difference in epigenetic clock index, as a proxy for the difference in mitotic age between the two samples.

Multiple tumor samples from the same patient provide a unique opportunity to assess the internal validity of the epigenetic clock. Indeed, paired samples should be epigenetically more related-via their most recent common ancestor cell-than samples from different patients. In a cohort of 8 women with multi-focal breast cancer, ^31^ we found that the within-patient correlations of the clock set fCpG sites were higher (median, 0.72) than the between-patient correlations (median, 0.10;P = 3×10^−6^, Wilcoxon rank-sum test; Fig. 7A). The same held true for a cohort of 18 patients with paired primary tumors and lymph node metastases ^32^ (median, 0.82 vs. 0.14, P = 5×10^−13^; Fig. 7B) and a subset of 22 patients with paired primary tumors and metastases (including lymph node and distant metastases) from the AURORA US Metastasis Project ^33^ (median, 0.73 vs. 0.08,P = 2×10^−26^; Suppl. Figure S4).

The two cohorts of patients with paired primary and metastasis samples ^32,33^ allowed us to test two additional properties of the fCpG clock. First, assuming that each metastasis is seeded by a single cell from the primary tumor, synchronous metastases should be younger than their matched primaries. Indeed, the epigenetic clock measures the age of the metastasis relative to the seeding event, which occurred after initiation of the primary tumor. Consistent with this prediction, in 36/40 patients, we found the metastases to have a lower epigenetic clock index compared to their matched primaries (Fig. 7C). This provides direct support for our interpretation of the epigenetic clock index as a proxy measure for mitotic age.

Second, the timing of metastatic dissemination relative to the primary tumor’s age is expected to impact the epigenetic similarity of the two samples: if the metastasis is seeded early during primary tumor growth (i.e., similar $c_{\beta }$ values), the β-values of the two samples are expected to be closely related (Figs. 7D-E) because the metastasis seeding cell came from a mostly homogenous population; conversely, if the metastasis is seeded late (i.e., different $c_{\beta }$ values), the β-values are expected to differ more substantially (Fig. 7F) because the seeding cell came from a heterogenous population. Corroborating this hypothesis, and consistent with a corresponding simulation of metastatic seeding based on the oscillator model (Fig. 7G), we found a negative correlation between mitotic age difference and β-value similarity (Fig. 7H).

### Quantifying mitotic tumor age

**Figure 8.**
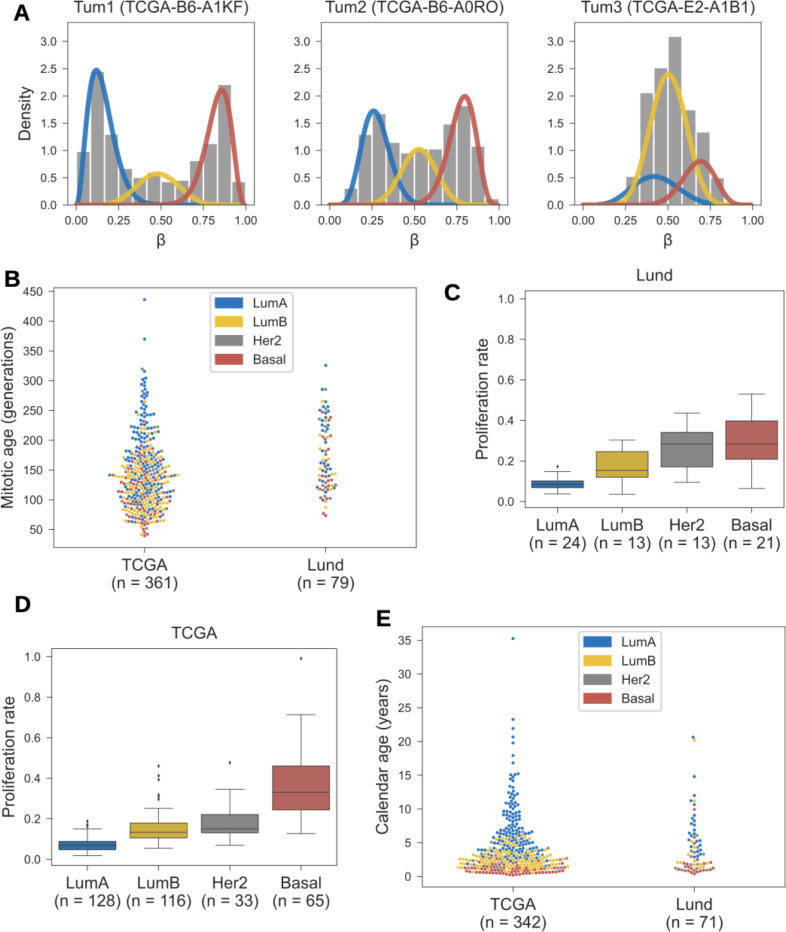
Estimating tumor age. **(A)** The empirical β-value distributions of the 500 fCpG sites in the clock set are decomposed into three peaks corresponding to initially unmethylated (blue), hemi-methylated (yellow), and fully methylated (red) sites. **(B)** Mitotic tumor age (the number of generations) is shown for the TCGA and Lund cohorts and colored by molecular subtype. These estimates are based on the peak locations (panel A) and a (de-)methylation rate of $\text{μ}=2\cdot 10^{-3}$, per cell division and per allele, which yields a mean tumor age of 3 calendar years (see panel E). **(C)** For the Lund cohort, tumor-specific proliferation rates are estimated using the measured fraction of cells in S-phase and shown by molecular subtype. **(D)** For the TCGA cohort, proliferation rates are estimated using S-phase fractions predicted based on gene expression, using a model trained on the Lund cohort. **(E)** Calendar age of tumors in the TCGA and Lund cohorts, colored by molecular subtype.

So far, we have used the epigenetic clock index $c_{\beta }$ as a correlate of mitotic tumor age. To derive quantitative estimates of each tumor’s mitotic and calendar ages, we proceeded as follows (see Methods for details). First, we invoked the mathematical oscillator model (Fig. 1A) to relate mitotic tumor age to the measured β-values of fCpG sites in the clock set. Next, we decomposed each tumor’s empirical fCpG β-value distribution into three groups (Fig. 8A): originally unmethylated fCpGs (left peak in the histogram), originally hemi-methylated fCpGs (middle peak), and originally methylated fCpGs (right peak). Finally, we combined the peak location in each group with the oscillator model to infer the estimated mitotic age of the tumor (Fig. 8B).

Finally, we combined tumor-specific estimates of mitotic age (Fig. 8B) and proliferation rate (Figs. 8C-D) to derive tumor-specific estimates of calendar age. Anchoring the median tumor age at a consensus estimate of 3 years (see Methods), the distribution of calendar ages across the TCGA and Lund cohorts ranged from 0.2 to 35.2 years, with an interquartile range of 1.5 to 5.6 years (Fig. 8E). There were notable differences in median tumor calendar ages by molecular subtype, ranging from 1.0 years in basal cancers to 6.5 years in Luminal A cancers (Suppl. Table S3).

### Adjusting for tumor purity

Bulk samples contain a mixture of tumor and stroma. Because the epigenetic clock index exhibited correlations with tumor purity as measured by the consensus purity estimate ^34^ (CPE; R=−0.67; Suppl. Figure S1A), we restricted our analyses to samples of high tumor purity (CPE ≥ 0.6). Nevertheless, we cannot rule out that the observed variability in β-value distributions among the selected fCpG sites-which are used to estimate mitotic tumor age-were at least partially driven by the methylation patterns of admixed non-epithelial cells. If this is the case, then, e.g., the immune pathway enrichment of older tumors (Fig. 6A) may be confounded by the presence of non-epithelial cells that alter the measured β-value distribution.

To adjust for possible confounding by tumor purity, we derived purity-adjusted β-values for the tumor cells by modeling the measured methylation as a mixture of tumor and stroma methylation, see Methods for details. The resulting purity-adjusted epigenetic clock index $c_{\beta }^{a}$ exhibited a lower correlation with tumor purity (R=−0.22; Suppl. Figure S1B) and was lower than the unadjusted epigenetic clock index $c_{\beta }$ (Suppl. Figure S1C).

When replacing the unadjusted epigenetic clock index with the purity-adjusted version, the strength of correlations between markers of tumor aggressiveness and younger mitotic age remained unaltered (Suppl. Table S1, Suppl. Figure S2, Suppl. Figure S3). Individual immune genes and the extent of immune infiltration remained associated with older mitotic age, although the correlations were attenuated (Suppl. Table S1, Suppl. Table S2). While the immune pathways were no longer enriched in older tumors (Suppl. Figure S3), there was still a positive correlation between the fraction of T cells and mitotic age (Suppl Table S2).

## DISCUSSION

In this study, we developed an epigenetic clock to measure the age of newly diagnosed breast cancers. Measuring epigenetic entropy among neutrally fluctuating CpG (fCpG) sites, the clock tracks mitotic tissue age on a time scale of years and, thus, provides higher temporal resolution compared to previous tissue clocks. Based on standard methylation arrays, it has the potential to be a novel marker of aggressiveness and prognosis in early-stage breast cancer.

Once a patient is diagnosed with breast cancer, the tumor’s mitotic age encodes valuable prognostic information. Intuitively, a slow-growing tumor that takes a long time to reach the threshold of detection is more likely to have a good prognosis compared to a fast-growing tumor that quickly expands into a detectable mass. Our analyses corroborate this hypothesis by revealing that mitotically younger tumors were enriched for features of tumor aggressiveness and predictors of poor outcome, including genomic instability, higher grade, and basal molecular subtype. ^24,25^ This property of the epigenetic clock is quite remarkable given that its constituent fCpG sites were selected from non-functional regions of the genome, on the basis of simple statistical properties of their β-value distributions.

Beyond prognostication, the clock holds promise in risk-stratified screening approaches. The efficacy of breast cancer screening critically depends on the sojourn time, that is the time window during which the tumor is asymptomatic but mammographically detectable. If the sojourn time is short, early detection is unlikely even under frequent screening; if it is long, some cancers will be overdiagnosed. ^35^ Sojourn time estimates are usually obtained by fitting natural history models to population data, yielding indirect, population-averaged estimates. Our approach, in contrast, allows for direct and individual-level characterization of tumor age, which provides a lower bound for the sojourn time. Assuming an overall median time to detection of 3 years in our cohort, the time to detection in luminal A cancers (6.5 years) was substantially longer compared to that in Luminal B (2.4 years) and basal (1.0 years) cancers. These estimates are consistent with the observation that interval cancers are enriched for more aggressive subtypes compared to screen-detected cancers, ^36^ and highlight opportunities for data-driven personalization of screening schedules.

The epigenetic clock also provides an opportunity to quantify the evolutionary-ecological pressures that shape the temporal landscape of breast cancers. Indeed, because most tumors are of comparable size at the time of diagnosis, mitotic age is related to the effective growth rate: tumors that reach the detection threshold at a younger age have a higher effective growth rate compared to tumors that reach the threshold at a higher mitotic age. Our analyses characterized the effective growth rate of breast cancers as a competition between tumor-intrinsic growth potential (e.g., proliferation) and microenvironmental pressures (e.g., surveillance by immune cells). ^37^ According to this model, highly proliferative tumors that successfully evade the immune system are detected at a younger age compared to less proliferative lesions subject to continuous immune control.

Our study has several limitations. First, because tumor age is not observable in practice, a direct validation of the clock is not possible. Nevertheless, we note that the clock correctly classified the age ordering of primary tumors and metastases in 36 of 40 patients. Second, the epigenetic clock index was correlated with sample purity, which suggests the latter may be a confounder in our analyses. To mitigate this risk of bias we systematically repeated all analyses using purity-adjusted methylation values; while some of the associations were attenuated, the overall qualitative conclusions remained unchanged. To address the potential confounding of age estimates by tumor purity, single cell methylation data is needed. Third, estimation of mitotic tumor age was based on a simple mathematical model of (de-)methylation dynamics. In future work, this approximation can be refined using more sophisticated simulation-based models that account for the underlying population dynamics, including cell proliferation and death, and possibly selection.

How long a newly diagnosed breast cancer has been growing is generally considered a known unknown. Here we revisited this assumption and developed a new way to infer tumor age using standard methylation arrays. While developed specifically for breast cancer, the approach can be generalized to any cancer type and, as such, provides a scalable technology to characterize the temporal landscape of oncology.

## METHODS

### TCGA Cohort.

Of the 1,085 invasive breast cancers from female patients in The Cancer Genome Atlas (TCGA), ^38^774 had available methylation array data (Infinium HumanMethylation450 BeadChip, Illumina, San Diego, CA, USA). After excluding 138 tumors of low tumor content (consensus purity estimate ^34^ [CPE] < 0.6) and one sample each from two patients with two primary samples, the remaining 634 tumors were used to select the ensemble of 500 fCpG sites. Finally, after excluding 10 tumors with ≥ 5% missing clock set fCpG measurements and 100 tumors with a histology code other than *infiltrating duct carcinoma*, the analytic cohort consisted of 400 tumors. The following variables were retrieved: patient age at diagnosis; tumor histology; T stage (subsetted to T1, T2, T3, and T4); summary stage (subsetted to stages I, II, or III). For all 400 patients with invasive ductal carcinoma, gene expression quantification (RNA-seq) and copy number segment data were available as well; when > 1 measurement was available, one was selected at random. All clinical and sequencing data were retrieved from the Genomic Data Commons (GDC; https://gdc.cancer.gov) using the R package *TCGAbiolinks* (version 2.25.3).

### Lund cohort.

We retrieved publicly available methylation array data (Infinium HumanMethylation450 BeadChip) from 181 primary breast cancers in the Southern Sweden Breast Cancer Group tissue bank at the Department of Oncology and Pathology, Skåne University Hospital (Lund, Sweden) and the Department of Pathology, Landspitali University Hospital (Reykjavik, Iceland). ^20^ The data were obtained through the Gene Expression Omnibus (GSE75067). Because calculation of the purity metric CPE requires gene expression, somatic copy-number, and immunohistochemistry in addition to methylation data, we instead assessed tumor purity using the leukocyte unmethylation percentage (LUMP) value. A tumor’s LUMP value is calculated as the average β-value among 44 specific CpG sites, divided by 0.85; we found the LUMP value to be strongly correlated with CPE (R=0.86). After excluding samples of low purity (LUMP < 0.6; n=35), the remaining 146 samples all had ≤ 5% missing clock set fCpG measurements. After exclusion of non-ductal histology (n=48) we ended up with an analytic cohort of n =98. The following variables were retrieved: patient age at diagnosis; tumor grade; tumor size; molecular subtype (PAM50); fraction of genome altered (FGA); expression of a mitotic checkpoint gene module,^39^ fraction of cells in S-phase (flow cytometry).

### Normal breast tissue cohort.

We obtained publicly available methylation array data (Infinium HumanMethylation450 BeadChip) from 100 normal breast tissue samples in the Susan G. Komen Tissue Bank ^17,40^ (GSE88883). We excluded samples of low purity (LUMP < 0.6), resulting in a cohort of 79 normal breast tissue samples used for identifying fCpG sites.

### Multiple sample cohorts.

We retrieved publicly available methylation array data (Infinium HumanMethylation450 BeadChip) from four cohorts with paired tumor samples. The first cohort consisted of 8 breast cancer patients with multiple primary samples (GSE106360). ^21^ Only samples from the 5 patients (2 patients with 5 samples each; 3 patients with 3 samples each) who had not received neoadjuvant therapy were used. Because LUMP values were highly variable, we did not apply any purity filtering. The second cohort consisted of 10 patients diagnosed with multi-focal breast cancer (GSE39451). ^31^ For each patient, methylation array data from 2 foci were available, and we only included the 8 patients where both samples were of sufficient purity (LUMP ≥ 0.6). The third cohort consisted of paired primary and lymph node metastasis samples from 44 patients (GSE58999). ^32^ Only patients where both samples were of sufficient purity (LUMP ≥ 0.6) were included (n=18). The fourth cohort, from the AURORA US Metastasis Project, consisted of primary and metastasis samples taken from 55 patients with metastatic breast cancer. In our analysis, we included only patients for whom at least one primary and one metastasis sample of sufficient purity (LUMP ≥ 0.6) were available (n=22) (GSE212370).^33^ Only patients with at least one primary and one metastasis sample of sufficient purity (LUMP ≥0.6) were included (n=22). When more than one primary or metastasis sample was available, the one with the highest LUMP value was selected.

### Selection of fluctuating CpG (fCpG) sites.

First, we identified CpG sites on the HumanMethylation450 BeadChip that correspond to functional regions of the genome. To this end, we identified sites that were associated with regulatory features or genes in one or both of the official annotation files of the Infinium HumanMethylation450 and MethylationEPIC bead chip arrays (https://support.illumina.com). After exclusion of such functional sites, a total of 86,099 CpG sites without functional annotation remained. Next, we sought to identify CpG sites with balanced methylation and demethylation rates, defined as having an average methylation content (β-value) between 0.4 and 0.6 in both the TCGA cohort (N=634) and the normal breast tissue cohort (N=79). CpG sites with ≥20 missing values in either cohort were excluded from this selection process. In the last step, we ranked all balanced CpG sites by their β-value variance among tumors in the TCGA cohort and selected the 500 most variable fCpGs to define the clock set C. Based on the clock set, each tumor was assigned an epigenetic clock index $c_{\beta }=1-s_{\beta }$ where $s_{\beta }$ is the standard deviation of the β values in the clock set.

### Gene expression analyses.

For tumors in TCGA, relative gene expression levels were taken as the mean-centered, log2(x+1) transformation of the reported transcript per million (TPM) intensities. Among the 60,616 RNA transcripts recorded in TCGA, only those classified as protein-coding genes by the HUGO Gene Nomenclature Committee ^41^ were included in subsequent analyses (n=18,910). Expression of MCM was calculated as the average relative expression in the 6-gene family MCM2–7, and expression of a *mitotic checkpoint* gene module ^39^ was calculated as the average relative expression of the genes included in the module. Molecular subtyping was based on the PAM50 algorithm ^42^ as implemented in R package *Genefu*. ^43^ For tumors in the Lund cohort, identical gene expression and molecular subtyping analyses had previously been reported, ^39^ thus enabling a direct comparison between tumors in the TCGA and Lund cohorts.

### Pathway enrichment analyses.

For the TCGA cohort, we performed a gene set enrichment analysis (GSEA) using the software package *GSEA* ^44,45^ to identify *Hallmark* gene sets that are correlated with the epigenetic clock index $c_{\beta }$. The analysis was performed using the Pearson correlation to rank individual genes; phenotype-permutation-based P values and false-discovery rate (FDR) Q values were computed using 1,000 permutations. All other inputs were kept at their defaults.

### CIBERSORTx.

To assess the immune cell composition within the tumor microenvironment, we employed CIBERSORTx using the LM22 signature matrix and batch correction. ^30^ Briefly, RNA-seq data from the TCGA tumor samples were uploaded to the CIBERSORTx web portal, where gene expression profiles were deconvoluted to estimate the absolute scores for 22 distinct immune cell types. The analysis was performed with the default parameters, including 100 permutations for statistical significance assessment. For reporting of results the 22 distinct cell types were then collapsed into six mutually exclusive categories: B cells, macrophages, mast cells, myeloid cells, natural killer (NK) cells, and T cells.

### Copy-number analyses.

For tumors in TCGA, copy-number (CN) data consisted of specified chromosomal regions of equal CN, the ${log}_{2}\;\left(\frac{x}{2}\right)$-transformed CN, and the number of probes. We converted these values to absolute copy numbers and determined each segment to have either a copy number gain (segment mean ≥ 2.5), a copy number loss (segment mean ≤ 1.5), or no change (1.5 < segment mean < 2.5). The fraction of the genome altered by copy number gains and losses were each calculated for every tumor by dividing the number of probes affected by gains and losses, respectively, by the total number of probes. The total fraction of the genome altered by copy number alterations (FGA) was then calculated as the sum of these two values. For tumors in the Lund cohort, the same approach had previously been used to compute FGA, ^2^ thus enabling direct comparison between tumors in the TCGA and Lund cohorts.

### In silico model of tumor growth and fCpG dynamics.

To simulate the dynamics of fCpG sites in a growing tumor, we used a discrete-time birth-death process. Starting with a single founding tumor cell, the population is updated in time intervals of one day, at which time each cell either divides, dies, or remains unchanged with probabilities α,λ, and 1−α−λ, respectively. Upon cell division, each allele in each cell changes its methylation state with probability μ. We tracked an ensemble of 90 fCpG sites, assuming independent (de-)methylation dynamics. Unless otherwise specified, the following parameters were used: α=0.17 (the estimated mean proliferation rate in the TCGA-Lund combined cohort, see below for details), λ=0.15 (to reach a population of 10^9^ cells in 3 years, or, $(1+\alpha -\lambda {)}^{3\cdot 365}\approx 10^{9}$), and μ=0.002 (the estimated flip rate in the combined cohort, see below for details).

### Tumor specific proliferation rates.

For tumors in the Lund cohort, tumor specific proliferation rates $\alpha_{i}$ were estimated based on the reported fraction $f_{i}$ of cells in S-phase as $\alpha_{i}=f_{i}/T_{S}$, where $T_{S}$ is the average time spent in S-phase (see Supplementary Methods for details). We assumed $T_{S}$ to equal 12.7 hours, based on an average across five cancer cell lines. ^46^ Because $f_{i}$ is not reported in the TCGA cohort, we used the Lund data to develop a predictive model of S-phase fraction using an elastic net model. As candidate predictors, we included FGA, LUMP value, and average gene expression levels within each of the following gene modules: ^39^ mitotic checkpoint (see above), immune response, stroma, mitotic progression, early response, steroid response, basal, and lipid. The model was fit to the Lund cohort tumors using cross-validation for hyperparameter optimization, and then applied to TCGA tumors to predict tumor-specific S-phase fractions $f_{i}$ and proliferation rates $\alpha_{i}$.

### Tumor age estimation.

To estimate tumor mitotic and calendar ages from the empirical β-value distributions, we proceeded in two steps. In the first step, we decomposed each tumor’s empirical β-value distribution into three groups, or “peaks”, of fCpG sites: the originally unmethylated fCpG sites (left peak), the originally hemi-methylated fCpG sites (middle peak), and the originally methylated fCpG sites (right peak). We achieved this by fitting a mixture model of three Beta distributions to the β-values of the 500 fCpG sites in the clock set using the R package BetaModels (version 0.5.2). To improve convergence of this method, sites with extreme β-values (β>0.98 or β<0.02) were removed before fitting the mixture model (a total of 69 and 442 sites were thus removed in the TCGA and Lund cohorts). In preparation of the next step, we determined the mode of each Beta component in the mixture as the location of the corresponding peak. At this point we excluded tumors with a middle peak location outside the interval [0.4,0.6] because this suggests a bias in the (de-)methylation rates and thus violates a basic assumption of the fCpG dynamics in the clock set (44 and 12 tumors were excluded in the TCGA and Lund cohorts, respectively). In the second step, we used the stochastic oscillator model (Fig. 1A) to relate the empirical peak location to the approximate age of the tumor. Because this step requires knowledge about the unknown stochastic (de-)methylation rate, we constrained the overall calendar age distribution across the Lund and TCGA cohorts to have a median of 3 years, which corresponds to the mean sojourn time in breast cancer. ^4,47^ See Supplementary Methods for details.

### Purity adjusted analyses.

Acknowledging the correlation between the epigenetic clock index $c_{\beta }$ and tumor purity, we derived a purity-adjusted epigenetic clock index $c_{\beta }^{a}$ and repeated relevant correlation analyses with $c_{\beta }^{a}$ instead of $c_{\beta }$. Because the epigenetic clock index was derived from the distribution of β-values of fCpG sites, we performed the purity adjustment at the level of β-values. For this, we assumed that the measured β-value at site $i\left(\beta_{i}^{m}\right)$ could be decomposed as a weighted sum of β-values of the tumor $\left(\beta_{i}^{t}\right)$ and the immune component $\left(\beta_{i}^{s}\right)$,

$$\beta_{i}^{m}=p\beta_{i}^{t}+(1-p)\beta_{i}^{s},$$

where p is the sample purity as measured by CPE. To estimate $\beta_{i}^{s}$ we combined the CIBERSORTx decomposition of the stroma (see section CIBERSORTx) with β-values of its constituent cells $\left(\beta_{k}^{c}\right)$ to obtain

$$\beta_{i}^{s}=\sum k\in LM22w_{i,k}\beta_{k}^{c},$$

where $w_{i,k}$ is the fraction of cell type k (in the LM22 signature) in tumor sample i. The $\beta_{k}^{c}$ were estimated using published cell-type specific methylation values.^48^ Finally, the purity adjusted β-values were obtained by solving Eq. (1) for $\beta_{i}^{t}$ and truncating values below 0 and above 1 (necessary for < 5.7% of the adjusted β-values).

### Statistical analyses.

Correlations between two continuous variables were calculated using the Pearson correlation coefficient. The medians of continuous variables were compared using a two-sided Wilcoxon rank-sum test at significance level of 0.05. For each variable, tumors with missing values of that variable were excluded. All analyses and visualizations were performed in Python (3.9.19) and R (version 4.3).

## Supplementary Material

Supplement 1

## Data Availability

All data used in this work are publicly available. Computer code used to generate the results is available at https://github.com/danmonyak/EpiClockInvasiveBRCA (MIT License).
