# Supplementary material for: Mapping the Temporal Landscape of Breast Cancer Using Epigenetic Entropy
Source: Res Sq. 2024 Oct 28:rs.3.rs-5119308. Preprint. [Version 1] doi: 10.21203/rs.3.rs-5119308/v1 (PMC11581123; doi:10.21203/rs.3.rs-5119308/v1)
Supplement: Supplement 1 [file NIHPPRS5119308V1-supplement-1.pdf]

Supplementary Files

This is a list of supplementary files associated with this preprint. Click to download.

- MolClockSupplmentaryv3.docx
- SupplementaryMethodsv3.pdf
